# Editorial: Current advances in genetic presentations of dementia and aging, volume II

**DOI:** 10.3389/fnagi.2023.1202532

**Published:** 2023-05-31

**Authors:** Yuzhen Xu, Ulises Gomez-Pinedo, Jun Liu, Daojun Hong, Jun Xu

**Affiliations:** ^1^Department of Rehabilitation, The Second Affiliated Hospital of Shandong First Medical University, Taian, China; ^2^Laboratory of Neurobiology, Department of Neurology, Institute of Neurosciences, IdISSC, Hospital Clínico San Carlos, Universidad Complutense de Madrid, Madrid, Spain; ^3^Department of Neurology, The Second Affiliated Hospital, Guangzhou Medical University, Guangzhou, China; ^4^Department of Neurology, The First Affiliated Hospital of Nanchang University, Nanchang, China; ^5^Department of Neurology, Beijing Tiantan Hospital, Capital Medical University, Beijing, China; ^6^China National Clinical Research Center for Neurological Diseases, Beijing Tiantan Hospital, Capital Medical University, Beijing, China

**Keywords:** genetic, aging, dementia, advance, Alzheimer's disease

Dementia associated with causative genes refers to a group of inherited disorders that cause progressive cognitive decline and dementia. These disorders are caused by mutations in certain genes that are involved in the normal function of the brain (Loy et al., 2014). The most common dementia associated with the causative gene is Huntington's disease. Aging is a natural process that affects all organisms, and it is a major risk factor for many diseases, including dementia. As we age, the structure and function of the brain change, and this can lead to cognitive decline and an increased risk of developing dementia (Singh et al., 2019). Some forms of genetic presentations of dementia are associated with accelerated aging and an earlier onset of cognitive decline. Similarly, mutations in certain genes can lead to accelerated aging and an earlier onset of cognitive decline. However, it is important to note that not all forms of genetic presentations of dementia are associated with accelerated aging, and the relationship between genetics, aging, and dementia is complex and not fully understood.

This topic aims to address the relationship between dementia associated with causative genes and aging, including the mechanism of action of various dementia risk genes during aging, identification of aging biomarkers associated with dementia associated with causative genes and strategies for aging prevention and treatment associated with dementia The topic will provide a comprehensive overview of the aging risk genes associated with causative genes and strategies to prevent and treat aging. This publication focuses on Alzheimer's disease (AD), neuronal intranuclear inclusion disease (NIID), type 2 diabetes mellitus (T2DM)-related cognitive impairment, Schizophrenia (SCZ), and co-morbid RNA-binding proteins (RBPs) of glioma and cerebral ischemia. This topic will provide potential new therapeutic strategies for aging-related dementia associated with causative genes.

AD is the most common type of dementia attributed to aging. The ε4 allele of the apolipoprotein E (APOE) gene is recognized as a strong genetic risk factor for AD. However, the role of cerebral blood flow (CBF) in cognition-related brain regions in mediating the association of APOE with cognition is unknown. The clinical study of Wang et al. proposed for the first time that CBF based on arterial spin labeling (ASL) technology could partially mediate the correlation between APOE genotype and cognition. Neurotrophic factors and their receptors have long been promising targets for the treatment of AD. The results of animal experiments by Wei et al. showed that the MET protein showed an age-dependent progressive decrease in the early stage of AD, and affected the activity of its ligand hepatocyte growth factor (HGF), suggesting that the decrease of the HGF/MET signaling pathway may be a potential cause of AD. One of the pathogenic mechanisms. Heart fatty acid binding protein (HFABP) is a regulatory factor in lipid metabolism and is considered to be a novel biomarker involved in the pathogenesis of AD. Fu et al. found that the level of HFABP in cerebrospinal fluid was correlated with the classic AD pathological marker p-Tau, which may affect the longitudinal changes of cognitive function by mediating the level of p-Tau. Visuospatial impairment is common in AD patients. Tokushige et al. investigated whether gaze exploration patterns during visual tasks could help predict cognitive decline in the early stages of AD.

Unlike AD, NIID, first reported in 1968, is a rare neurodegenerative disease that affects cognitive function. The current diagnosis of NIID relies on CGG repeat expansion in the 50 UTR of the *NOTCH2 NLC* gene, or p62-positive intranuclear inclusions in skin biopsy. Skin biopsies are limited in scope due to their novelty. The study of Zhou et al. suggested that urine cytology is a sensitive and reliable non-invasive method for diagnosing NIID, although its accuracy is not as good as that of skin biopsy.

T2DM is an important risk factor affecting cognitive function in the elderly. Liu G. et al. found that T2DM may be related to dementia by affecting the volume of the fourth ventricle in a cross-sectional study of Chinese elderly communities, but this conclusion still needs further large-scale and multi-center verification. Zhang Y. et al. used astragaloside IV (AS-IV) to intervene in T2DM model rats, and found that its therapeutic effect may be achieved by regulating the Nrf2/Keap1/HO1/NQO1 pathway to reduce oxidative stress and neuroinflammation. In addition to AS-IV, quercetin, a traditional Chinese medicine, has recently been considered to prevent diabetic cerebrovascular endothelial cell injury by targeting VCAM1 (Huang et al., 2022).

SCZ is a multi-etiological mental illness that can cause cognitive impairment and neuropsychological disorders. It currently affects about 20 million people worldwide and is one of the leading causes of disability. The results of bioinformatics-based research by Zhang C. et al. showed that NEUROD6, NMU, PVALB, and NECAB1 may be potential biomarkers for predicting SCZ. In addition, Lin et al. also explored the comorbid RBPs of glioma and ischemic stroke, namely POLR2F, DYNC1H1, SMAD9, TRIM21, BRCA1, and ERI1. Among them, upregulated SMAD9 is associated with dementia, while downregulated POLR2F is associated with aging-related hypoxic stress. In the future, RBP is expected to be used as a comorbidity biomarker of glioma and ischemic stroke to guide clinical prevention and treatment.

ASL and urine cytology techniques offer no innovative options for early diagnosis and treatment of AD and NIID. The mechanisms of HGF/MET, HFABP in the pathogenesis of AD have been further explored. T2DM-related cognitive impairment has been at the forefront of research, and the detection of Nrf2/Keap1/HO1/NQO1 signaling pathway and fourth ventricular volume has helped to unravel the pathogenic mechanisms of the disease. Interestingly, bioinformatics has an increasingly prominent role in predicting neurological diseases such as SCZ, glioma and cerebral ischemic comorbidity RBPs. In addition, gaze exploration patterns may also be important indicators of response to early AD. In conclusion, the genetic manifestations of dementia are complex and current studies are insufficient to present the full picture of the disease, especially its concomitant aging manifestations deserve further investigation.

## Author contributions

YX drafted the manuscript. All authors contributed to the article and approved the submitted version.
